# Comment on “Do we need flexible machine-learning algorithms to assess the effect of long-term exposure to fine particulate matter on mortality?”

**DOI:** 10.1097/EE9.0000000000000407

**Published:** 2025-07-14

**Authors:** Nicholas Williams, Kara E. Rudolph, Iván Díaz

**Affiliations:** a Department of Epidemiology, Mailman School of Public Health, Columbia University, New York, New York; b Division of Biostatistics, Grossman School of Medicine, New York University, New York, New York

## To the Editor:

Studies comparing the performance of statistical estimators are essential for choosing the most appropriate estimator for an analysis.^1^ Chen et al.^2^ compare a parametric and nonparametric estimator for assessing the effect of long-term PM_2.5_ exposure on mortality. They conclude that the nonparametric estimator offers “minimal” benefit over the parametric one. However, the authors’ approach has two key flaws that preclude the ability to draw valid conclusions. We explain why and propose solutions below.

We also point to a recent study of ours that sought to quantify the extent to which using more flexible, “fancy,” nonparametric estimators that use machine learning for model fitting outperformed simple, parametric regression estimators, if at all, in finite samples.^1^ We evaluated performance across not one, but thousands of simulated data scenarios. We found that nonparametric estimators substantially outperformed parametric estimators in settings characterized by nonlinearities and interactions. In simple settings or in small sample sizes of N = 100, performance was similar.^1^

### Key issue 1: Inaccurate definition of bias and variance

What Chen et al.^2^ refer to as “bias” and “variance” do not correspond to statistical definitions of these concepts. The bias of an estimator is defined as its average across random draws of datasets from a population minus the true value of the estimand. In contrast, the quantity Chen et al.^2^ refer to as “bias” is the difference between a single estimate and a hypothetical value. Similarly, the variance of an estimator is defined as the average squared difference between an estimator and its mean across random draws of datasets from a population. The quantity Chen et al.^2^ refer to as “variance” is a point estimate of the estimator variance from a single dataset.

Estimating bias and variance usually requires a simulation study that (1) draws multiple datasets from a data-generating mechanism that reflects the real data, and (2) by the data-generating mechanism being known, bestows knowledge of the true value of the statistical estimand.^3^ Thus, Chen et al.^2^ did not—and could not given their methodology—compute the actual bias and variance of the estimators.

A solution would be to use appropriate simulation methods for comparing estimators. For example, plasmode simulations generate simulated data with a known effect while preserving the complex relationships among covariates in the observed data.^4,5^ Alternatively, the authors could have used the general approach that we used and described above.^1^

### Key issue 2: Consideration of a nonpathwise differentiable estimand

This second key issue is technical, and not widely appreciated in epidemiology. However, it has a serious implication, which is that the resulting TMLE estimate reported by Chen et al.^2^ does not have valid standard errors. The authors’ statistical estimand is the risk difference in mortality under a hypothetical threshold intervention, where PM_2.5_ exposure is set to 8.8 µg/m^3^ for observations exceeding that level, compared to leaving exposure unchanged. This statistical estimand is nonsmooth (intuitively, because the intervention sets a continuous exposure to a fixed value). In statistical jargon, the estimand is not pathwise differentiable in the nonparametric model. Lack of pathwise differentiability means that it is not possible to use an estimator in the nonparametric model, such as TMLE,^6^ and still rely on standard statistical theory to support uncertainty quantification.

A solution would be to redefine the statistical estimand. For instance, we could evaluate the effect of a hypothetical intervention in which the natural value of PM_2.5_ is reduced by 20% if above 8.8 µg/m^3^ or use a stochastic threshold intervention where exposures above the threshold get randomly allocated to an interval under it.^6–8^

## Conflicts of interest statement

The authors declare that they have no conflicts of interest with regard to the content of this report.
